# Immunity and Extracellular Matrix Characteristics of Breast Cancer Subtypes Based on Identification by T Helper Cells Profiling

**DOI:** 10.3389/fimmu.2022.859581

**Published:** 2022-06-20

**Authors:** Yan Zhou, Qi Tian, Huan Gao, Lizhe Zhu, Ying Zhang, Chenchen Zhang, Jiao Yang, Bo Wang

**Affiliations:** ^1^ Department of Medical Oncology, the First Affiliated Hospital of Xi’an Jiaotong University, Xi’an, China; ^2^ Department of Breast Surgery, the First Affiliated Hospital of Xi’an Jiaotong University, Xi’an, China; ^3^ Department of Gastroenterology, the First Affiliated Hospital of Xi’an Jiaotong University, Xi’an, China; ^4^ Department of Clinical Laboratory, the 940th Hospital of Joint Logistics Support Force of Chinese People’s Liberation Army, Key Laboratory of Stem Cells and Gene Drug in Gansu Province, Lanzhou, China; ^5^ Center for Translational Medicine, the First Affiliated Hospital of Xi’an Jiaotong University, Xi’an, China; ^6^ Key Laboratory for Tumor Precision Medicine of Shaanxi Province, Xi’an, China

**Keywords:** breast cancer, T helper cell (Th), tumor microenvironment, extracellular matrix, tumor immunity

## Abstract

**Background:**

The therapeutic effect of immune checkpoint inhibitors on tumors is not only related to CD8+ effector T cells but also sufficiently related to CD4+ helper T (T_H_) cells. The immune characteristics of breast cancer, including gene characteristics and tumor-infiltrating lymphocytes, have become significant biomarkers for predicting prognosis and immunotherapy response in recent years.

**Methods:**

Breast cancer samples from The Cancer Genome Atlas (TCGA) database and triple-negative breast cancer (TNBC) samples from GSE31519 in the Gene Expression Omnibus (GEO) database were extracted and clustered based on gene sets representing T_H_ cell signatures. CIBERSORT simulations of immune cell components in the tumor microenvironment and gene set enrichment analyses (GSEAs) were performed in the different clusters to verify the classification of the subtypes. The acquisition of differentially expressed genes (DEGs) in the different clusters was further used for Gene Ontology (GO) and Kyoto Encyclopedia of Genes and Genomes (KEGG) analyses. The clinical information from different clusters was used for survival analysis. Finally, the surgical tissues of TNBC samples were stained by immunofluorescence staining and Masson’s trichrome staining to explore the correlation of T_H_ cell subtypes with extracellular matrix (ECM).

**Results:**

The breast cancer samples from the datasets in TCGA database and GEO database were classified into T_H_-activated and T_H_-silenced clusters, which was verified by the immune cell components and enriched immune-related pathways. The DEGs of T_H_-activated and T_H_-silenced clusters were obtained. In addition to T_H_ cells and other immune-related pathways, ECM-related pathways were found to be enriched by DEGs. Furthermore, the survival data of TCGA samples and GSE31519 samples showed that the 10-year overall survival (*p*-value < 0.001) and 10-year event-free survival (*p*-value = 0.162) of the T_H_-activated cluster were better, respectively. Fluorescent labeling of T_H_ cell subtypes and staining of the collagen area of surgical specimens further illustrated the relationship between T_H_ cell subtypes and ECM in breast cancer, among which high T_H_1 infiltration was related to low collagen content (*p*-value < 0.001), while high T_H_2 and T_reg_ infiltration contained more abundant collagen (*p*-value < 0.05) in TNBC. With regard to the relationship of T_H_ cell subtypes, T_H_2 was positively correlated with T_reg_ (*p*-value < 0.05), while T_H_1 was negatively correlated with both of them.

**Conclusions:**

The immune and ECM characteristics of breast cancer subtypes based on T_H_ cell characteristics were revealed, and the relationship between different T_H_ cell subsets and ECM and prognosis was explored in this study. The crosstalk between ECM and T_H_ cell subtypes formed a balanced TME influencing the prognosis and treatment response in breast cancer, which suggests that the correlation between T_H_ cells and ECM needs to be further emphasized in future breast cancer studies.

## Introduction

The emergence of immunotherapy brought solid tumors to a new era (1), especially in breast cancer, which has the highest incidence (2). Currently, many clinical studies related to immune checkpoint inhibitors (ICIs) mainly focus on the regulation of CD8+ cytotoxic T cells (CTLs) on the tumor immune microenvironment (TIME) and the possible mechanism of immune-related targets, for example, IMpassion 130 (3), KEYNOTE-119 (4), and the FUTURE trial (5) for advanced breast cancer and KEYNOTE-173 (6) and IMpassion 031 (7) for early breast cancer. In reality, as the principal component of tumor-infiltrating lymphocytes (TILs), T lymphocytes play a key role in the occurrence and development of breast cancer, especially in triple-negative breast cancer (TNBC) (8, 9). Among TILs, not only CTLs but also CD4+ helper T (T_H_) cells directly or indirectly exert protumorigenic or/and antitumorigenic immune effects by affecting other immune cells, especially CTLs, through the inflammatory molecules secreted by different subtypes of T_H_ cells and the accommodation of signal transduction (10, 11). It is precisely the various and interlaced immunoregulatory properties of T_H_ cells that make the optimization of immunotherapy based on them more clinically significant. Based on previous studies (12–15), the conference on T_H_1 (T helper type 1), T_H_2 (T helper type 2), T_H_17 (T helper type 17), T_fh_ (T follicular helper), and T_reg_ (CD4+ regulatory T) cells as a group of CD4+ T_H_ cells that are independent and have a chain reaction with TILs is very meaningful to avoid tumor immune escape and improve the efficacy of immunotherapy for breast cancer.

Apart from immune cells, cancer-associated fibroblasts (CAFs) are also prominent components of the tumor microenvironment (TME) (16, 17), and the activation of cytokines such as interleukin-1 (IL-1) and IL-6 and immune-related pathways such as the Janus kinase-signal transducer and activator of transcription (JAK-STAT) and nuclear factor kappa-B (NF-κb) pathways plays an important role in the generation and recruitment of CAFs (18, 19). In fibrosis-related diseases, including tumors, each subtype of T_H_ cells has different regulatory effects on fibroblast-induced collagen synthesis through inflammatory factors, including interferon-γ (IFN-γ) (20). In turn, CAFs also regulate the activation and function of T_H_ cells by secreting cytokines and chemokines (16, 21). The extracellular matrix (ECM) structured through collagen produced by CAFs in the TME not only affects the differentiation of T cells but also affects the spatial distribution of T cells (22) to modulate antitumorigenic immunity by influencing the dialog between T cells and tumor cells.

Based on TIME-related characteristics as biomarkers for breast cancer prognosis and prediction of immunotherapy efficacy, many studies (23–26) have proposed prognostic models and detailed breast cancer typing in recent years. A total score of the immune microenvironment as indicators of the classification for the data samples was employed in most of them (27, 28). In contrast, this study will utilize the genetic traits of T_H_ cells, such as heterogeneous and particular cells, and explore the characteristics of the TIME and ECM in breast cancer to put forward a reference for the breakthrough of immunotherapy in the field of T_H_ cells.

## Materials and Methods

### Datasets and Clinical Samples

RNA-sequencing data of 1,097 breast cancer patients from The Cancer Genome Atlas (TCGA) database and the corresponding clinical data were extracted. Microarray gene expression data using the Affymetrix U133A array of 64 TNBC patients from GSE31519 were obtained from the National Center for Biotechnology Information (NCBI) in the Gene Expression Omnibus (GEO) Database, in which clinical survival data are available. The data from the above public databases were used for cluster analysis based on T_H_ cell characteristics and for differential analysis and survival analysis among different clusters.

Formalin-fixed paraffin-embedded (FFPE) surgical tissue sections (4 µm thick) from 30 TNBC patients admitted to the Department of Medical Oncology, The First Affiliated Hospital of Xi’an Jiaotong University during 2016–2020 were used for immunofluorescent staining and Masson’s trichrome staining, which is for the analysis of different T_H_ subtypes and collagen content in the TIME of TNBC patients. The detailed clinical information of patients is described in **Supplementary Table S1**, in which disease-free survival (DFS) was defined as the time from surgery to the occurrence of the first metastasis.

### Identification of Subtypes Based on T_H_ Cell Characteristics in Breast Cancer

Consensus cluster analysis was employed to identify breast cancer subtypes based on T_H_ cell characteristics obtained from the single-sample gene set enrichment analysis (ssGSEA) (29, 30). The gene set representing five T_H_ cell subtypes, including T_H_1, T_H_2, T_H_17, T_fh_, and T_reg_ cells, was used for clustering, as shown in **Supplementary Table S2**. The “ConsensusClusterPlus” package (http://www.bioconductor.org/) was used to divide the datasets with duplicate samples removed (1,090 in the TCGA dataset and 64 in the GSE31519 dataset, as shown in **Supplementary Tables S3**, **S4**, respectively) into k=2–9 subgroups followed by hierarchical agglomerative consensus, and optimal clustering was obtained by comparing the consensus matrix and cumulative distribution function (CDF) value.

### Evaluation of TIME in Breast Cancer Samples From Datasets

The CIBERSORT deconvolution algorithm was used to estimate the fraction of 22 immune cell types in each sample to evaluate TIME in breast cancer (31), which was calculated *via* the online calculator (https://cibersort.stanford.edu/). The CIBERSORT results were filtered by *p*-value < 0.05 to obtain more accurate prediction results, and the samples upon the filter conditions were employed in subsequent differential analysis between different clusters (846 in TCGA dataset and 63 in GSE31519 dataset as shown in **Supplementary Tables S5**, **S6**, respectively). Principal component analysis (PCA) was used to distinguish immune cell components between different clusters through dimensionality reduction, pattern recognition, and exploratory visualization.

### Gene Set Enrichment Analysis

GSEA was performed to compare the gene expression of different clusters by using GSEA version 4.1.0 provided by the Broad Institute (http://software.broadinstitute.org/gsea/index.jsp). Kyoto Encyclopedia of Genes and Genomes (KEGG) pathway enrichment analysis obtained through gene sets from the Broad Institute (http://ftp.broadinstitute.org://pub/gsea/gene_sets/c2.cp.kegg.v6.2.symbols.gmt) was used to determine the enriched pathways of clusters. In GSEA, the significance of each pathway was classified by a threshold of false discovery rate (FDR) q-value <0.05.

### Extraction of Differentially Expressed Genes, Gene Ontology, and KEGG Pathway Enrichment Analysis of DEGs

T_H_-related differentially expressed genes (DEGs) were extracted and analyzed using the R packages “Limma,” “Impute,” and “EdgeR” through the gene expression profiles of different clusters. DEGs were filtered by logFCfilter=2 or 1 and fdrFilter=0.05. Gene Ontology (GO) analysis was conducted for the high-throughput annotation of biological functions (BPs), cellular components (CCs), and molecular function (MF) of the DEGs between different clusters, while KEGG analysis was conducted for the molecular and pathway levels of the DEGs. The R packages “DOSE,” “ClusterProfiler,” “Org.Hs.eg.db,” and “Enrichplot” were used in the GO and KEGG pathway enrichment analyses of DEGs (*p*-value < 0.05).

### Tissue Immunofluorescent Staining and Masson’s Trichrome Staining

The FFPE tissue sections were deparaffinized to water using graded ethanol-dimethylbenzene, employed in ethylenediaminetetraacetic acid (EDTA) buffer (pH 8.0, ZHHC, PI001) for 15 min to recover the antigen, incubated in Triton X-100 (ZHHC, PI024) for 5 min to implement a permeable membrane, and sealed with bovine serum albumin (BSA). To label the T_H_1, T_H_2, and T_reg_ subsets of the TIME in TNBC, sections were incubated overnight at 4 °C with primary antibodies against T-bet (Abcam, ab150440, 1:30), GATA-3 (Abcam, ab199428, 1:30), and CD25 (Abcam, ab231441, 1:50) and CD4 (Proteintech, 67786-1-Ig, 1:200). The sections were washed with phosphate-buffered saline (PBS) and incubated with the secondary antibody (Proteintech, SA00003-1 and SA00009-2, 1:50) at room temperature for 60 min. The nuclei were stained with 4,6-diamidino-2-phenylindole (DAPI) (ZHHC, CD110) for 3 min. The stained cells were observed, and images were acquired by a confocal laser scanning microscope (Leica Company) in at least 10 fields per section. For Masson’s trichrome staining, the deparaffinized sections were analyzed with a Masson’s trichrome stain kit (Solarbio, G1340) and were observed, and images were acquired by a Leica scanning optical microscope after dehydration, clearing, and mounting.

### Survival Analysis

For plotting Kaplan–Meier (K–M) curves of TCGA and GSE31519 clusters based on T_H_ cell characteristics, the R packages “survival” and “survminer” were used, while the log-rank test was employed to assess the significance of overall survival (OS) and event-free survival (EFS) differences. Similarly, K–M survival analysis and log-rank tests were conducted to compare the differences in DFS between the different groups of T_H_ cell subtypes in 30 TNBC samples.

### Statistical Analysis

The statistical analyses of data from the public database were performed using R software (http:///www.r-project.org/) and Bioconductor (http://bioconductor.org/). To compare any two groups of datasets in the study, the Wilcoxon test was conducted using R software. The R packages “pheatmap,” “ggplot2,” and “vioplot” were used to show the differential results in the study. The statistical analyses of 30 TNBC samples collected by us were performed using GraphPad Prism 8.0.0 (GraphPad Software Inc., San Diego, CA, USA). The non-parametric independent sample *t*-test was used for the difference in the different groupings of T_H_ cell subtypes, and Pearson’s correlation was used for the correlation between different T_H_ cell subtypes, in which the data were all normally distributed. All *p-*values were bilateral, and a *p*-value < 0.05 was considered statistically significant.

## Results

### Identification of Subtypes by T_H_ Cells and the Corresponding TIME in Breast Cancer

**Figure 1:**
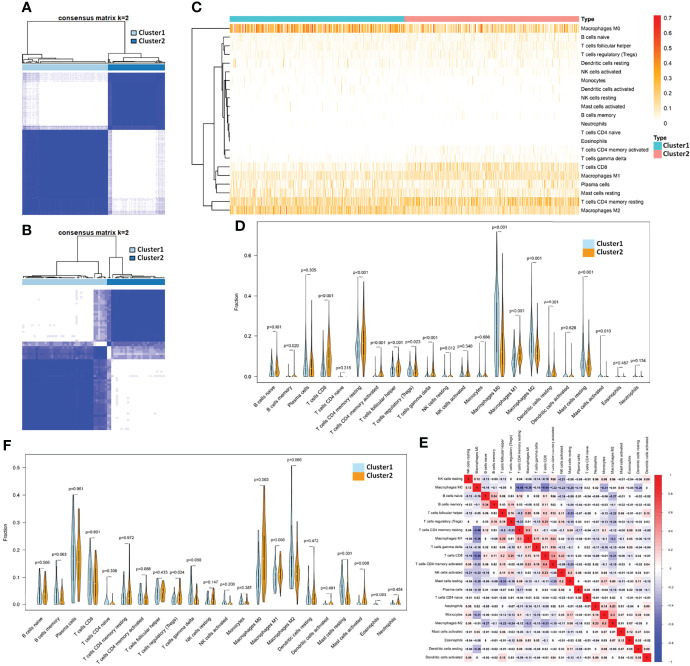
Identification of subtypes by T_H_ cells and the corresponding TIME in breast cancer. **(A)** The consensus matrix when k =2 through cluster analysis based on the expression of T_H_ cell-related gene characteristics in 1,090 samples from TCGA database and **(B)** 64 samples from GSE31519 dataset. Heatmap **(C)** and violinplot **(D)** of the fraction of 22 kinds of immune cells in the TIME of the two clusters obtained from the TCGA database. **(E)** Heatmap of the correlation of 22 immune cell components obtained from the TCGA database. **(F)** Violinplot of TIME in the two clusters from the GSE31519 dataset.

The transcriptome and clinical data of 1,097 breast cancer samples from TCGA database were extracted, and the mRNA expression of 1,090 breast cancer samples after the removal of duplicate samples was analyzed. According to the expression of T_H_-cell-related gene characteristics, 1,090 samples were collected for cluster analysis. From the clustering results of k = 2–9, it can be found that when the samples were clustered into two clusters (k = 2, Cluster 1 included 654 samples and Cluster 2 included 436 samples, see **Supplementary Table S3** for details), the consensus matrix had a relatively average distribution, less matrix overlap, and a smoothly decreasing CDF value (**Figure 1A**; **Supplementary Figures S1A–D**). In addition, we extracted the transcriptomic data of 64 TNBC samples from the GSE31519 dataset of the GEO database for the same cluster analysis as above (**Figure 1B**; **Supplementary Figures S2A–D**), which was still optimal when the samples were divided into two clusters (k=2, Cluster 1 included 38 samples and Cluster 2 included 26 samples; see **Supplementary Table S4** for details).

The deconvolution algorithm CIBERSORT was used to verify the immune microenvironment of the two clusters obtained from the TCGA dataset by simulating the fraction of 22 kinds of immune cells in the TIME of each sample (see **Supplementary Table S5** for details). Differential analysis of immune cell components showed that Cluster 2 had more resting memory CD4 T cells, activated memory CD4 T cells, T_fh_ cells, gamma delta T cells (*p*-value < 0.001), and T_reg_ (*p* value = 0.023) than Cluster 1. Therefore, Cluster 1 was defined as a T_H_-silenced cluster, and Cluster 2 was defined as a T_H_-activated cluster. Except for the above T_H_ cells, the T_H_-activated cluster showed significantly more infiltration containing CD8 T cells, naive B cells, memory B cells, M1 macrophages, and resting dendritic cells and less infiltration of M0 macrophages, M2 macrophages, and resting mast cells (**Figures 1C,D**). In the TCGA samples, the correlation of 22 immune cell components can be seen in **Figure 1E**, in which CD8 T cells have a strong positive correlation with T_reg_ cells and activated memory CD4 T cells, and M1 macrophages were positively correlated with T_fh_ cells and resting memory CD4 T cells, while M2 macrophages showed the opposite correlation. Similarly, CIBERSORT was used to verify the TIME of the samples in the GSE31519 dataset (see **Supplementary Table S6** for details), from which we found that T_fh_ cells, T_reg_, and M1 macrophages were more abundant in Cluster 2 than in Cluster 1, while M2 macrophages and resting mast cells were less abundant, and the differences in other immune cells were not statistically significant (**Figure 1F**). Therefore, Cluster 1 in the GSE31519 dataset was still defined as the T_H_-silenced cluster, and Cluster 2 was defined as the T_H_-activated cluster.

### Verification Towards the Characteristics of the Two Clusters Identified by T_H_ Cells

**Figure 2:**
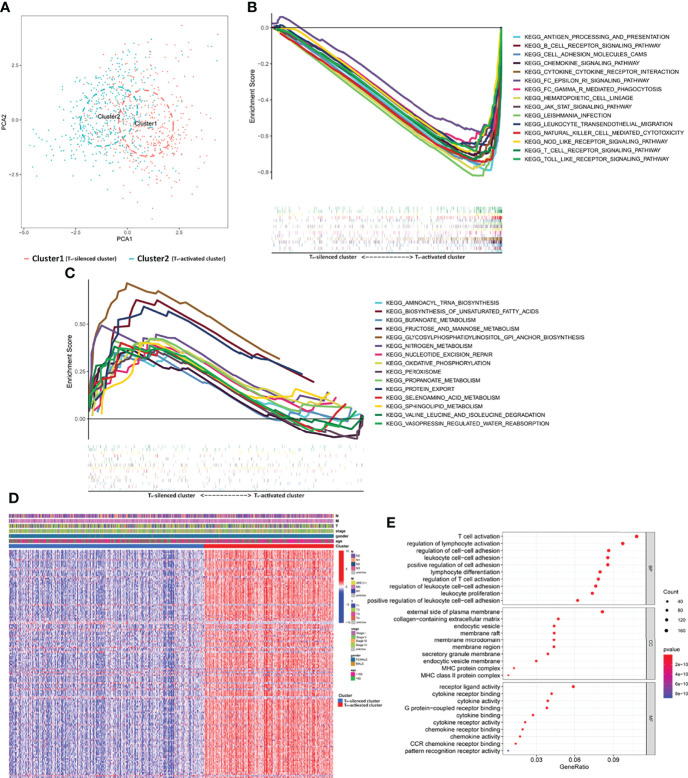
The gene profile characteristics of two clusters identified by T_H_ cells from TCGA breast cancer samples cued the correlation between T_H_ cells and ECM in the TIME. **(A)** PCA diagram of 1,090 samples classified into T_H_-silenced cluster and T_H_-activated cluster. **(B)** GSEA showed that the pathways of NES top 15 in the T_H_-activated cluster included abundant immune-related KEGG pathways, while **(C)** top 15 in the T_H_-silenced cluster included glucose metabolism- and fatty acid synthesis-related pathways. **(D)** The heatmap of differential expression of the DEGs in the two clusters (logFCfilter=2 and fdrFilter=0.05) and the distribution of clinical information. **(E)** GO enrichment analysis of the DEGs showed that collagen-containing ECM was enriched except for the immune-activated GO.

A total of 1,090 samples classified into T_H_-silenced clusters and T_H_-activated clusters were evaluated for immune cell components using PCA. The two clusters were clearly separated (**Figure 2A**). The transcriptome data were prepared for the KEGG pathway-related GSEA, in which the pathways of normalized enrichment score (NES) top 15 in the two clusters were selected for demonstration. Activation of immune-related pathways was enriched in the T_H_-activated cluster, including the T cell, B-cell receptor signaling, and cytokine and chemokine signaling pathways (**Figure 2B**). This further verified the stronger immunogenicity and immune activity of the samples in the T_H_-activated cluster than in the T_H_-silenced cluster. The pathways enriched in the T_H_-silenced cluster included glucose metabolism-, amino acid metabolism- and fatty acid synthesis-related pathways (**Figure 2C**).

### The Pathways Enriched by DEGs of Two Clusters Cued the Correlation Between T_H_ Cells and ECM

Samples from the two clusters of TCGA database were used for gene expression differential analysis. When logFCfilter=2 and fdrFilter=0.05, 148 DEGs were obtained (see **Supplementary Table S7** for details). The differential expression of these DEGs in the two clusters and the distribution of clinical information, including TNM stage, stage, age, and gender, are shown in **Figure 2D**. GO enrichment analysis (**Figure 2E**) showed that BP of the DEGs was most significantly related to the activation of T cells and differentiation and activation of lymphocytes, while the MF portion was also mostly cytokine related. In addition to the above manifestation that immune-activated GO is enriched with upregulation in T_H_-activated cluster versus T_H_-silenced cluster, collagen-containing ECM was enriched in the aspect of CC, indicating that synthesis of collagen and the construction of ECM may be correlated with T_H_ cell activation.

**Figure 3:**
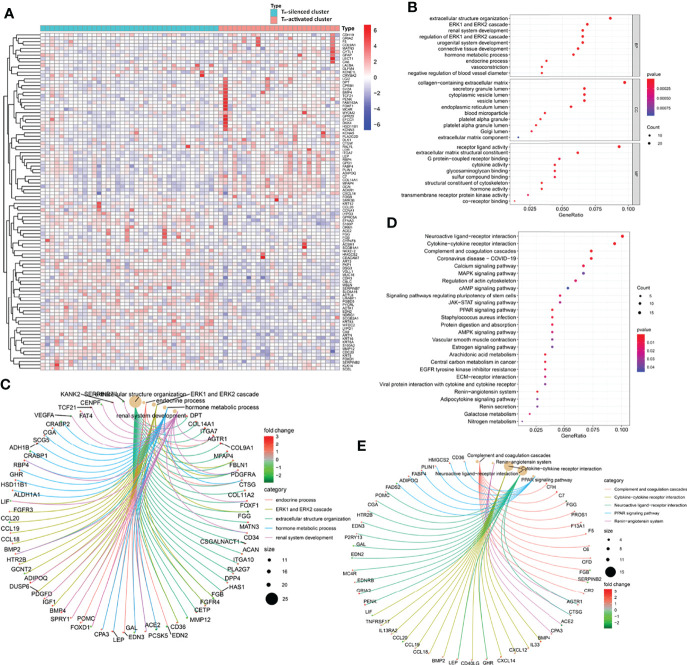
The gene profile characteristics of two clusters identified by T_H_ cells from GSE31519 TNBC samples cued the correlation between T_H_ cells and ECM in the TIME. **(A)** Heatmap of the differential expression of the 100 prominent DEGs between the two clusters from the GSE31519 dataset (logFCfilter=1 and fdrFilter=0.05). The bubble diagram **(B)** and circle diagram **(C)** showed that ECM-related pathways were enriched from GO enrichment analysis of the DEGs. The bubble diagram **(D)** and circle diagram **(E)** showed that cytokine–cytokine receptor interaction, JAK-STAT signaling pathway, and ECM–receptor interaction were enriched from KEGG enrichment analysis of the DEGs.

A total of 310 DEGs were obtained between the transcriptional data of the samples in the two clusters from the GSE31519 dataset (see **Supplementary Table S8**, logFCfilter=1 and fdrFilter=0.05). The up- and downregulated expression of the 100 prominent DEGs between the two clusters is shown in the heatmap in **Figure 3A**. Furthermore, GO enrichment analysis of DEGs showed that ECM-related pathways were enriched in parts of BP, CC, and MF by comparing the T_H_-activated cluster versus the T_H_-silenced cluster, while cytokine activity was enriched in MF (**Figures 3B, C**). The possibility that ECM is associated with T_H_ cells was reconfirmed by KEGG pathway enrichment analysis of these DEGs (**Figures 3D, E**), in which cytokine–cytokine receptor interaction and JAK-STAT signaling pathways related to immunity were enriched with upregulation, and ECM-receptor interaction was also enriched.

### The Correlation Between Different T_H_ Cell Subtypes and ECM

**Figure 4:**
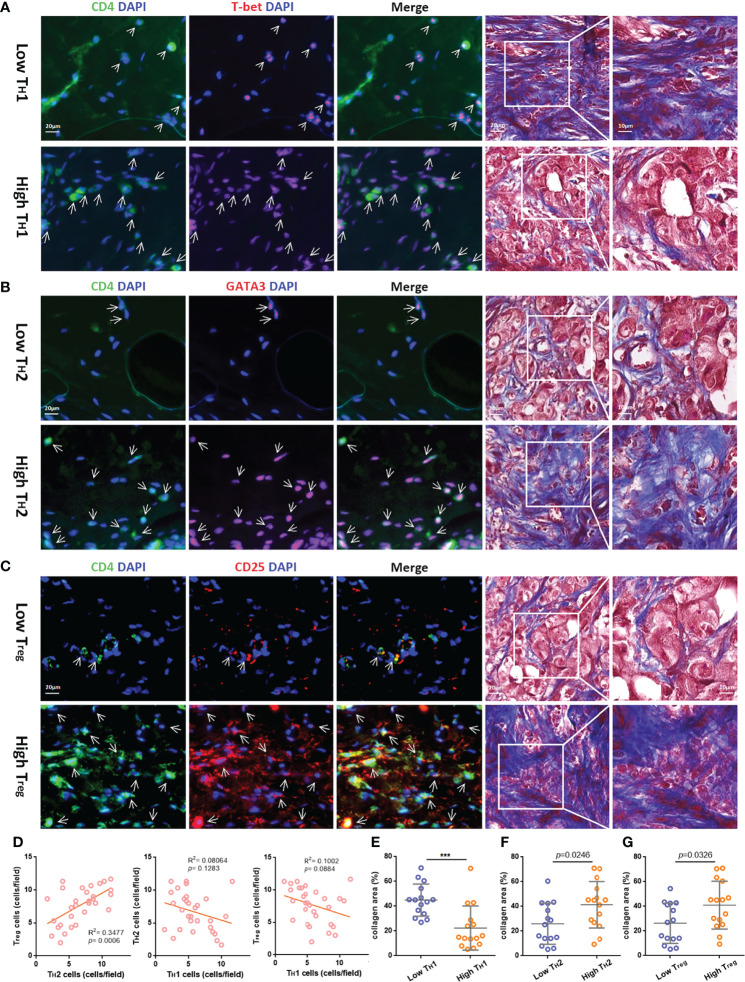
The relativity between different T_H_ cell subtypes and the correlation of different T_H_ cell subtypes and ECM in the TIME. Representative immunofluorescent staining for CD4 (green), T-bet, GATA3, or CD25 (red) and 4,6-diamidino-2-phenylindole (DAPI) (blue) (bar = 20 μm.) and corresponding Masson’s trichrome staining (bar = 10/20 μm.) in TNBC samples with low or high tumor-infiltrating T_H_1 **(A),** T_H_2 **(B)**, and T_reg_ cells **(C)**, which are indicated by arrows. **(D)** Pearson correlation analysis of the three subtypes of T_H_ cells. The *t*-test analysis of variance between the collagen area of two groups with low or high tumor-infiltrating T_H_1 **(E)**, T_H_2 **(F)**, and T_reg_ cells **(G)**. ***p value < 0.001.

To further explore the content of tumor-infiltrating T_H_ cell subtypes in breast cancer tissues, we performed immunofluorescence staining on paraffin tissue sections of primary foci from 30 TNBC patients, in which CD4^+^T-bet^+^ T_H_1, CD4^+^GATA3^+^ T_H_2, and CD4^+^CD25^+^ T_reg_ cells were labeled (see **Supplementary Table S1** for clinical information and corresponding T_H_ cell content of these patients). According to the different contents of T_H_1, T_H_2, and T_reg_ cells, 30 samples were divided into two groups for follow-up analysis (see **Supplementary Figures S3A–C** for the distribution after grouping of each T_H_ cell subtype content). To explore the correlation between different subtypes of tumor-infiltrating T_H_ cells and ECM in breast cancer, the collagen area in breast cancer tissues of grouping based on T_H_ cell subtypes was analyzed for difference (the collagen area of each sample was the average value of three random fields after Masson’s trichrome staining, as shown in **Supplementary Table S1**). The breast cancer tissues with higher T_H_1 cell content had less collagen distribution; for example, a low tumor-infiltrating T_H_1 cell sample had abundant collagen, while a high tumor-infiltrating T_H_1 cell sample had the opposite distribution, as shown in **Figure 4A**. In contrast, as shown in **Figures 4B, C**, breast cancer tissues with higher tumor-infiltrating T_H_2 cells and T_reg_ content were more collagen distributed. Furthermore, Pearson correlation analysis was conducted on the contents of the three subtypes of T_H_ cells, and it was found that the content of T_H_2 was positively correlated with T_reg_ (R^2 =^ 0.3477, *p*-value < 0.001), while T_H_1 was negatively correlated with T_H_2 and T_reg_ (*p*-value > 0.05), as shown in **Figure 4D** (the content of T_H_ cell subtypes at each data point was the average value of three random fluorescence staining fields). **Figures 4E–G** are the *t*-test analyses of variance between the two groups of T_H_1 grouping (*p*-value < 0.001), T_H_2 grouping (*p*-value = 0.0246), and T_reg_ grouping (*p*-value = 0.0326).

### The Prognosis Of Different Groupings Based on Total T_H_ Cells and T_H_ Cell Subtypes

**Figure 5:**
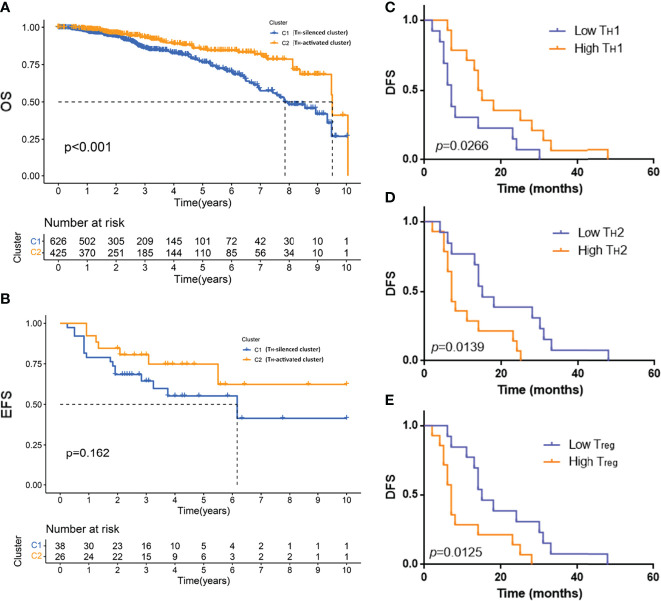
The prognosis of the T_H_-activated cluster and T_H_-silenced cluster in breast cancer and the prognosis of different groups based on T_H_ cell subtypes. Kaplan–Meier (K–M) survival analysis was employed for 10-year overall survival (OS) of 1,090 samples from the TCGA database **(A)** and the 10-year event-free survival (EFS) of 64 TNBC samples from the GSE31519 dataset **(B)**. K–M survival curve of disease-free survival (DFS) of 30 TNBC samples grouped by low or high tumor-infiltrating T_H_1 **(C)**, T_H_2 **(D)**, and T_reg_ **(E)** cells. Two-tailed log-rank *p*-values are shown.

To verify that the T_H_-activated cluster and T_H_-silenced cluster indicated significance for prognosis, K–M survival analysis was employed for samples from the public database. The 10-year OS of 1,090 samples from the TCGA database was compared in two clusters, of which the OS of the T_H_-activated cluster was longer (**Figure 5A**, *p*-value < 0.001). The 10-year EFS of 64 TNBC samples from the GSE31519 dataset was compared in two clusters, of which the EFS of the T_H_-activated cluster was longer (**Figure 5B**, *p*-value = 0.162).

Based on the fluorescence staining results of T_H_ cells of the 30 TNBC samples mentioned above, DFS of patients with different groupings of T_H_ cell subtypes was employed to plot the K–M survival curve. Patients with high T_H_1 cell infiltration had a better prognosis with longer DFS, as shown in **Figure 5C** (*p*-value = 0.0266), while DFS was shorter in patients with high T_H_2 and high T_reg_ cell infiltration, as shown in **Figures 5D, E** (T_H_2 grouping *p*-value = 0.0139, T_reg_ grouping *p*-value = 0.0125).

## Discussion

The predictive significance for treatment response and changes in tumor development of TILs as biomarkers have been an issue discussed in breast cancer as solid tumors. Many studies (32) have also classified breast cancer into high or low TIL subtypes to guide the treatment and prognosis of patients. In addition, a large number of studies (27, 33) have focused on the classification of breast cancer subtypes by immune score based on inflammatory factors and immune-cell-related genes in recent years. In the breast cancer subtype, TNBC was more widely treated with immunotherapy because of the emergence of effective biomarkers such as PD-L1 and well-founded classification of full inflamed (FI), stroma restricted (SR), margin restricted (MR), and immune desert (ID) subtypes (34). Therefore, in addition to all breast cancer samples of TCGA database, a TNBC dataset from GEO database was incorporated in this study, and the verification of T_H_ cell subtypes was also conducted in TNBC samples. In other solid tumors, 65 combinations of T-cell markers have been used as indicators for determining the generation and early metastasis of colorectal cancer (35), while immune-related genes have also been used as gene sets for subtyping squamous cell carcinoma and lymphoma (36, 37). Regarding the role of subtyping and predicting the prognosis of breast cancer based on T cells, a study (38) showed that the gene score based on CD8+ T cells was associated with survival, especially in TNBC. In this study, a breast cancer dataset and a TNBC dataset were both divided into T_H_-silenced cluster and T_H_-activated cluster based on the gene characteristics of T_H_ cells, among which two datasets had similar distributions of T_H_ cell-related gene characteristics. The T_H_-activated cluster was characterized by upregulated immune-related gene characteristics, more activated immune-related pathways, and better prognosis, suggesting that the employment of T_H_ cells as an independent biomarker for breast cancer could be achieved and have no less clinical significance than other prominent immune cell components, such as CTLs.

The role of T_H_ cells in the TIME is related to the inflammatory factors secreted by them, while these inflammatory factors cannot only affect other types of immune cells but also interact with each other among different T_H_ cell subtypes. As an example, IFN-γ secreted by T_H_1 can further induce the activation of STAT1 and STAT4 in T cells, thereby promoting T_H_1 differentiation with positive feedback and inhibiting T_H_2 and T_H_17 differentiation (39). Conversely, IL-4 secreted by T_H_2 can regulate T_H_2 differentiation with positive feedback while inhibiting T_H_1 differentiation (40), from which it can be found that the balance between IFN-γ and IL-4 is also the balance of T_H_1–T_H_2 in the TIME, corresponding to the balance of pro- and antitumorigenic immune effects. T_regs_ induced by transforming growth factor-β (TGF-β) in the TIME activate the STAT5 signaling pathway by binding IL-2 with high affinity through CD25 and exert opposite regulatory effects on T_H_1 and T_H_2 cells by secreting IL-10, IL-35 and TGF-β (41). In this study, correlation analysis of T_H_1, T_H_2 and T_reg_ in TIME of TNBC also showed that T_H_2 was positively correlated with T_reg_, while T_H_1 was negatively correlated with both of them, which further confirmed that a relationship network containing promotion and restriction was formed between T_H_ cell subtypes to achieve the balance of pro- and antitumorigenic immunity. In addition, a study (42) showed that different subtypes of peripheral blood T_regs_ in breast cancer patients have different effects on the secretion of intratumor T_H_1- and T_H_2-related inflammatory factors, which also suggests that it is necessary to pay attention to T_H_ cells in peripheral blood.

In this study, ECM-related pathways were enriched in the T_H_-activated cluster, and the relationships between different T_H_ cell subtypes and ECM were detected in clinical specimens. It was found that high T_H_1 infiltration was related to low collagen content, while TME with high T_H_2 and T_reg_ infiltration contained more abundant collagen in TNBC. A study (43) has shown that IFN-γ can upregulate the expression of matrix metalloproteinases (MMPs) that degrade ECM components, such as MMP-2, MMP-7, MMP-9, and MMP-13. Therefore, IFN-γ secreted by T_H_1 cells may be the possible cause of ECM remodeling, and the antagonistic relationship between T_H_2 and T_H_1 cells may also lead to the opposite effect on ECM. Furthermore, IL-13 secreted by T_H_2 is also considered to be a factor positively related to ECM formation in many fibrotic diseases (44). The effects of T_reg_ on fibrosis are not the same in different diseases, while studies (45) have shown that the decrease in IL-10 and TGF-β levels caused by T_reg_ depletion may be the crucial procedure leading to the reduction in fibrosis. In addition, a study (46) reported that the expression level of fibrosis-related transcription factors in tissue-resident T_reg_ cells was increased in renal fibrosis disease. In breast cancer, research on tissue-resident memory T (T_RM_) cells (47) as an emerging target in immunotherapy reveals that the combination of tissue-resident T_reg_ cells and fibrosis-related biomarkers has outstanding clinical significance. On the other hand, in terms of the effect of ECM on T_H_ cells in the TME, a study (48) found that the elimination of FAP+ CAFs *in vivo* can implement the polarization of T_H_2 cells to T_H_1 cells; moreover, in breast cancer, the CAF1-S1 subtype could achieve immunosuppression by recruiting and increasing the differentiation of CD4+CD25+ T_reg_ (49). Therefore, the crosstalk between ECM and T_H_ cell subtypes persistently accumulates, forming a balanced TME in breast cancer.

Similarly, a recent study (50) showing the association between clusters of CAFs and immunotherapy resistance by single-cell sequencing highlighted the positive feedback loop relationship between specific CAFs-S1 clusters and T_reg_ and revealed the correlation between different CAF clusters and CD8+ and CD4+ T cells, which indicates that the identification of specific clusters instructs treatment and prognosis in cancer. In reality, different T_H_ cell subtypes have different prognostic guidance for solid tumors, which has been proven by studies (51, 52) based on characteristic gene expression data from clinical samples and animal experiments. This study also found that DFS of 30 TNBC patients was longer under high T_H_1 infiltration, low T_H_2, and low T_reg_ infiltration. Accordingly, studies of ECM characteristics identifying different prognoses in breast cancer gradually appeared in 10 years, in which a stiff TME with abundant ECM indicating a poor prognosis was generally approved (53). In addition, different subtypes of TNBC have different prognoses and treatment strategies according to the spatial heterogeneity of CD8+TILs (54), which makes consideration of whether the distribution of CD4+ T_H_ cells in the TIME also has crucial clinical value. Correspondingly, the spatial distribution of T_H_ cells and ECM-related studies (22) further reminded us of the significance of T_H_ cell characteristics and ECM as a new combined biomarker for breast cancer. The intrinsic modulatory mechanism of cytokines secreted by CD4+ T_H_ cells influencing ECM remodeling would be our study field in the future, which is a crucial dimension to explain the correlation of the TME with prognosis in breast cancer and find potential combined therapies in the clinic.

## Conclusion

Overall, the immune and ECM characteristics of breast cancer subtypes based on T_H_ cell characteristics were revealed in this study by analyzing the datasets, and the relationship between different T_H_ cell subsets with ECM and prognosis were explored in clinical TNBC samples. The accumulation of crosstalk between ECM and T_H_ cell subtypes formed a balanced TME in breast cancer, which suggests that the combination of T_H_ cell characteristics and ECM as a new biomarker needs to be further emphasized in future breast cancer clinical studies.

## Data Availability Statement

The datasets presented in this study can be found in online repositories. The names of the repository/repositories and accession number(s) can be found in the article/**Supplementary Material**.

## Ethics Statement

The studies involving human participants were reviewed and approved by the Ethics Committee of the First Affiliated Hospital of Xi’an Jiaotong University. The patients/participants provided their written informed consent to participate in this study.

## Author Contributions

YaZ, QT, and BW contributed to the study design and performed the experiments. HG and LZ contributed to data collection. YiZ, QT, CZ, and JY performed statistical analysis and interpretation. YaZ and BW drafted the manuscript. All authors contributed to the article and approved the submitted version.

## Funding

This study was supported by the National Natural Science Foundation of China (No. 82002794), Key Research and Development Program of Shaanxi Province of China (No. 2015SF015 and 2019SF-147), and Institutional Foundation of the First Affiliated Hospital of Xi’an Jiaotong University (No. 2019ZYTS-13).

## Conflict of Interest

The authors declare that the research was conducted in the absence of any commercial or financial relationships that could be construed as a potential conflict of interest.

## Publisher’s Note

All claims expressed in this article are solely those of the authors and do not necessarily represent those of their affiliated organizations, or those of the publisher, the editors and the reviewers. Any product that may be evaluated in this article, or claim that may be made by its manufacturer, is not guaranteed or endorsed by the publisher.

## Supplementary Material

Supplementary Figure 1 Comparison of optimal cluster analysis for 1090 samples from TCGA database. The consensus matrix when k =3 **(A)** and k =4 **(B)** through cluster analysis based on the expression of T_H_ cell-related gene characteristics in 1090 samples from TCGA database. The cumulative distribution function (CDF) value **(C)** and relative change in the area under the CDF curve **(D)** of k =2-9.

Supplementary Figure 2 Comparison of optimal cluster analysis for 64 samples from the GSE31519 dataset. The consensus matrix when k =3 **(A)** and k =4 **(B)** through cluster analysis based on the expression of T_H_ cell-related gene characteristics in 64 samples from the GSE31519 dataset. The cumulative distribution function (CDF) value **(C)** and relative change in the area under the CDF curve **(D)** of k =2-9.

Supplementary Figure 3 The distribution of contents of T_H_ cell subtypes after grouping in 30 TNBC samples. The distribution of contents of T_H_1 **(A)**, T_H_2 **(B)** and T_reg_ **(C)** cells after low or high infiltrating grouping in 30 TNBC samples.
