# Supplementary figures and images for: Immunity and Extracellular Matrix Characteristics of Breast Cancer Subtypes Based on Identification by T Helper Cells Profiling
Source: Front Immunol. 2022 Jun 20;13:859581. doi: 10.3389/fimmu.2022.859581 (PMC9251002; doi:10.3389/fimmu.2022.859581)

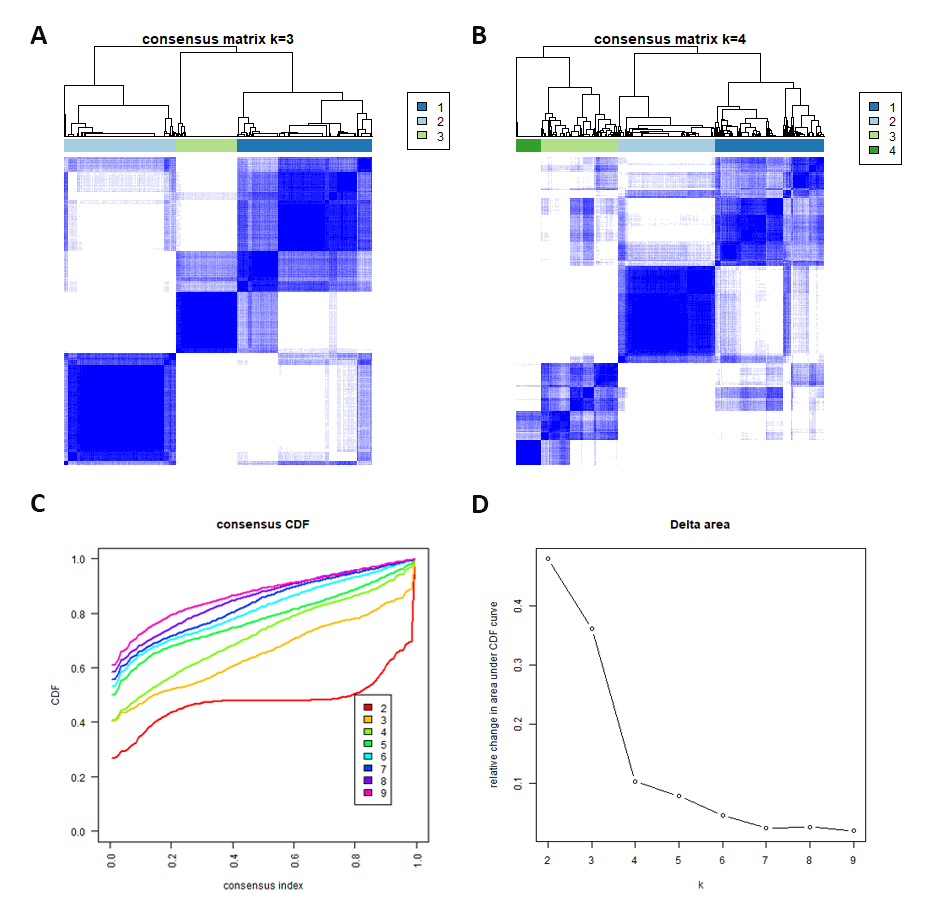

Supplement: Supplementary Figure 1 — Comparison of optimal cluster analysis for 1090 samples from TCGA database. The consensus matrix when k =3 (A) and k =4 (B) through cluster analysis based on the expression of TH cell-related gene characteristics in 1090 samples from TCGA database. The cumulative distribution function (CDF) value (C) and relative change in the area under the CDF curve (D) of k =2-9. [file Image_1.tif]

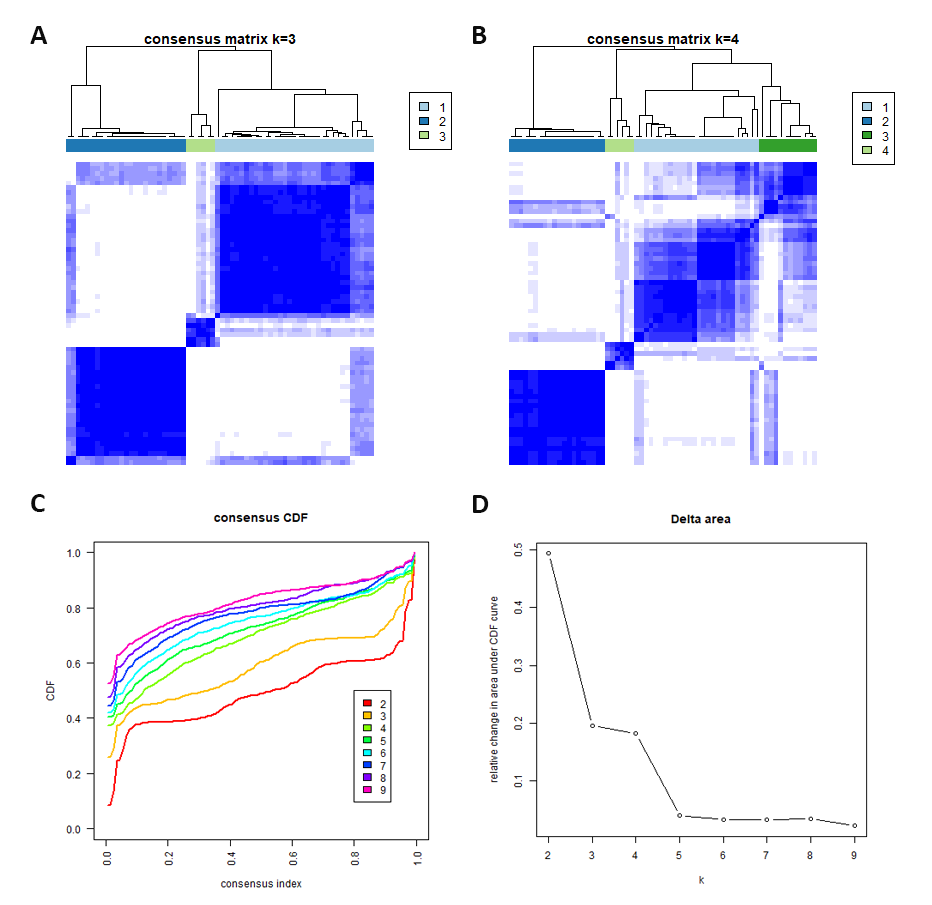

Supplement: Supplementary Figure 2 — Comparison of optimal cluster analysis for 64 samples from the GSE31519 dataset. The consensus matrix when k =3 (A) and k =4 (B) through cluster analysis based on the expression of TH cell-related gene characteristics in 64 samples from the GSE31519 dataset. The cumulative distribution function (CDF) value (C) and relative change in the area under the CDF curve (D) of k =2-9. [file Image_2.tif]

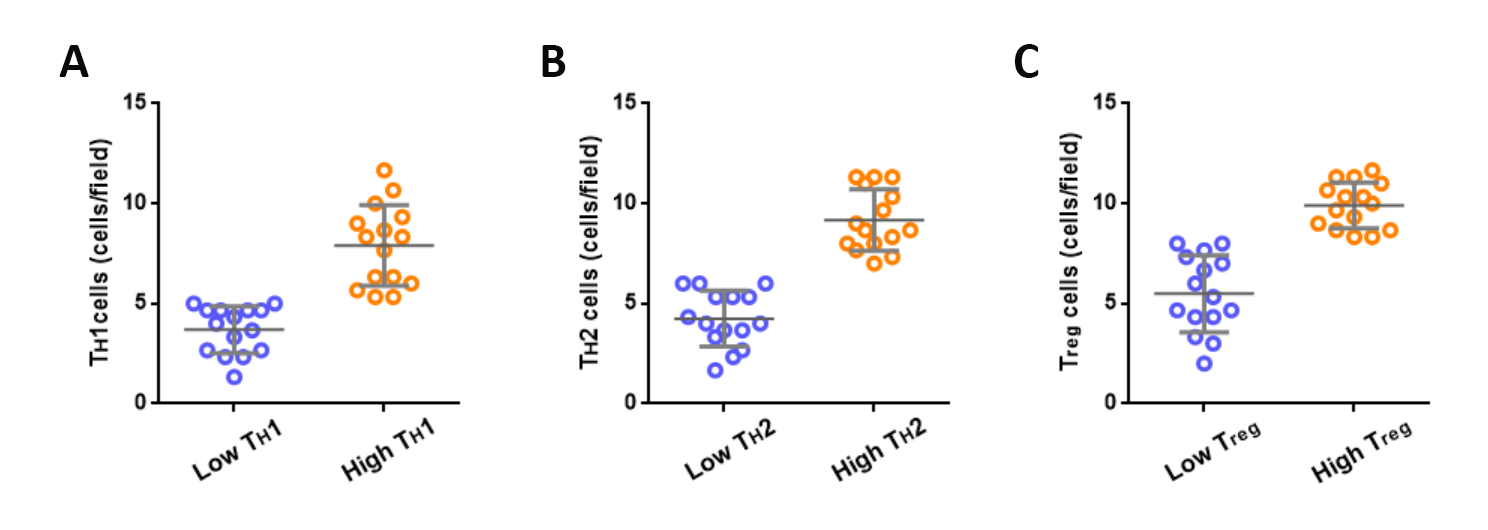

Supplement: Supplementary Figure 3 — The distribution of contents of TH cell subtypes after grouping in 30 TNBC samples. The distribution of contents of TH1 (A), TH2 (B) and Treg (C) cells after low or high infiltrating grouping in 30 TNBC samples. [file Image_3.tif]
